# GRIK1 promotes glioblastoma malignancy and is a novel prognostic factor of poor prognosis

**DOI:** 10.32604/or.2023.043391

**Published:** 2024-03-20

**Authors:** GUOQIANG HOU, XINHANG XU, WEIXING HU

**Affiliations:** 1 Department of Neurosurgery, First Affiliated Hospital of Nanjing Medical University, Nanjing, China; 2 Department of Neurosurgery, Renji Hospital, Shanghai Jiao Tong University, Shanghai, China

**Keywords:** Glioblastoma, GRIK1, Invasion, Proliferation, Prognosis

## Abstract

Primary tumors of the central nervous system (CNS) are classified into over 100 different histological types. The most common type of glioma is derived from astrocytes, and the most invasive glioblastoma (WHO IV) accounts for over 57% of these tumors. Glioblastoma (GBM) is the most common and fatal tumor of the CNS, with strong growth and invasion capabilities, which makes complete surgical resection almost impossible. Despite various treatment methods such as surgery, radiotherapy, and chemotherapy, glioma is still an incurable disease, and the median survival time of patients with GBM is shorter than 15 months. Thus, molecular mechanisms of GBM characteristic invasive growth need to be clarified to improve the poor prognosis. Glutamate ionotropic receptor kainate type subunit 1 (GRIK1) is essential for brain function and is involved in many mental and neurological diseases. However, GRIK1’s pathogenic roles and mechanisms in GBM are still unknown. Single-nuclear RNA sequencing of primary and recurrent GBM samples revealed that GRIK1 expression was noticeably higher in the recurrent samples. Moreover, immunohistochemical staining of an array of GBM samples showed that high levels of GRIK1 correlated with poor prognosis of GBM, consistent with The Cancer Genome Atlas database. Knockdown of GRIK1 retarded GBM cells growth, migration, and invasion. Taken together, these findings show that GRIK1 is a unique and important component in the development of GBM and may be considered as a biomarker for the diagnosis and therapy in individuals with GBM.

## Introduction

Primary tumors of the central nervous system encompass over 100 different histological types. The most frequent and lethal tumor of the central nervous system is glioblastoma, which is nearly impossible to completely remove surgically because of its propensity for growth and invasion [1–5]. The medium survival time of patients who suffer from GBM is still less than 15 months [6,7], although delightful development has been made in the treatment of GBM patients [8]. Thus, it is important to explore GBM biology and determine new molecular targets relevant to the therapeutic effect. According to recent studies, there are many factors associated with glioma cell growth and invasion, such as Vav guanine nucleotide exchange factor 3 (VAV3) [9], long noncoding RNA LINC01711 (lncRNA-LINC01711) [10], collagen alpha-2(I) chain (COL1A2) [11], NIMA-related kinase 2 (NEK2) [12], and RAN binding protein 10 (RANBP10) [13], etc.

Glutamate is the most widespread excitation neurotransmitter in the mammalian brain [14]. Both ionotropic (ligand-gated ion channels) and metabotropic (G protein-coupled receptors) glutamate receptors have been cloned [15]. With similarity in between nucleotide and amino acid sequences, at least six gene families have been identified as encoding ionotropic glutamate receptors, and there are also three families of NMDA receptors, two families of KA receptors, and one family of AMPA receptors [16]. Brain areas involved in learning and memory contain GluK1, a subunit of the glutamate ionotropic receptor kainate type gene (GRIK1) [17], and the gene’s precise location is 21q21.1-22.1 [16]. These ligand-gated ion channels are crucial for healthy brain function and play a significant role in various neurological and behavioral disorders [18]. The term “tumor microenvironment” (TME) describes a specific type of environment produced by cancer cells as well as numerous other cell types, including endothelial cells, fibroblasts, immune cells, and extracellular components. The intensive metabolic activity of cancer cells can alter the nutritional composition and structure of the cells and tissues that make up the TME to more efficiently meet the needs of the tumor cells for growth. Glutamate receptors are involved in the regulation of a range of cells in the TME, including immunological and cancer cells, as cell surface receptors [19,20].

The GRIK1 gene is known to be particularly implicated in schizophrenia within the glutamate system [21]. In addition, several recent reports have suggested that GRIK1 may be an important factor for predicting the progression or prognosis of colorectal carcinoma [22], and breast tumor [23], it is the most prevalent kinds of cancer in women and one of the main causes of cancer deaths [24], and the progression of breast cancer is also related to many elements, such as metastasis-related gene signature [25], and has_circ_0000069 [26]. A polymorphism (rs2832407) in GRIK1, which encodes the GluK1 kainate subunit, modifies topiramate-related reduction in heavy drinking [27,28], while a GRIKI polymorphism forecasts negative effects after topiramate therapy [28]. Meanwhile, polymorphisms in GRIK1 are associated with hepatitis B virus–related hepatocellular carcinoma (HCC) [29] and coronary artery aneurysm (CAA) formation in patients with Kawasaki disease (KD) [30]. Additionally, in healthy social drinkers, a GRIK1 mutation affects the subjective response to intravenous alcohol in a dose-related manner [31]. Although the research area of GRIK1 is already very extensive, the pathological functions and mechanisms of GRIK1 in GBM are still unclear.

Six samples of primary and recurrent GBM were gathered for the current study, and they were each examined using a single nuclear sequencing. GRIK1 levels significantly rose in recurring samples. Furthermore, not only in The Cancer Genome Atlas (TCGA) database but also in a variety of GBM samples, the prognosis of GBM patients was inversely correlated with the expression levels of GRIK1. Studies on the function of cells revealed that GRIK1 was essential for the invasive growth of GBM. Thus, we discovered a new and important element in the formation of GBM, and GRIK1 may be considered as a new target for the diagnosis and treatment of GBM.

## Materials and Methods

### Patients’ tissue samples

This study was approved by the Ethics Committee of Shanghai Jiao Tong University’s Renji Hospital (Institutional Review Board [IRB] number, RA-2022-032). All patients gave their informed consent, and the entire research was carried out in line with all applicable laws. Cancerous tissues were collected, and normal tissues were obtained from trauma patients. Sixty-five patients diagnosed with GBM were enrolled, and their follow-up data were collected. The age of GBM patients (36 men and 29 women) ranged from 22 to 76 years (Suppl. Table 1).

### Single-nuclear sequencing library preparation and data processing

Based on the tumor tissue samples gathered from the GBM patients who received treatment at Shanghai Jiao Tong University’s Renji Hospital, single-nuclear RNA sequencing was carried out. With consent and in accordance with the methodology approved by the IRB, the biopsy samples from the patients were collected for research. Libraries were created in accordance with the Chromium Next GEM Single Cell 3 Reagent Kits v3.1 manufacturer’s instructions. Cellular barcodes were demultiplexed, reads were mapped to the genome and transcriptome using the STAR aligner, reads were downsampled as necessary to generate normalized aggregate data across samples, and a matrix of gene counts *vs.* cells was created using the Cell Ranger software pipeline (version 3.1.0) provided by 10Genomics. Using the R package Seurat (version 3.1.1), we processed the unique molecular identification (UMI) count matrix. The bioinformatics analysis and sequencing were carried out by Oebiotech Co., Ltd. (Shanghai, China).

### Investigation of the TCGA database’s survival data

The TCGA provided information on the gene expression levels and related pathologies for GBM. Gene expression levels were divided into high and low levels. The Kaplan-Meier method was used to look into survival estimations and examine the survival disparity.

### Western blot

WB assays were done using rabbit anti-human GRIK1 (cat 25779-1-AP, Proteintech) and mouse anti-actin (cat ab8226, Abcam, Cambridge, UK) antibodies. RIPA buffer (cat P0013B Beyotime Biotechnology, Shanghai, China) with protease inhibitor complex (0-4693124001, Roche, CompleteMini) was used to extract the proteins from the samples. The lysates were divided into 10% SDS-PAGE gels (cat P0012AC, Beyotime Biotechnology, Shanghai, China). The isolated proteins were then put on PVDF membranes and blocked for an hour with 5% nonfat milk at 37°C. The membranes were incubated with primary antibody, namely, rabbit anti-GRIK1 antibody (1:1000) (cat 25779-1-AP, Proteintech, Wuhan, China) for 8 h at 4°C and then cleaned with TBS (pH 7.4) which added 0.1% Tween-20 and then wash them three times. The membranes were incubated with mouse anti-actin antibody (1:10,000) for 1 h. The secondary HRP-conjugated antibodies were utilized, and bands were detected using an ECL kit (cat P0018S, Beyotime Biotechnology, Shanghai, China).

### RT-qPCR

TRIzol reagent (16096020, Thermo Fisher Scientific, USA) was used to extract RNA. Reverse transcription of RNA was done by HiScript II RT SuperMix (cat R223-01, Vazyme, China). Forward primer for GRIK1 was 5′-AGTGCCATAGACATAAGCCATG-3′, while the reverse primer for GRIK1 was 5′-AATCCTTCCTCAAGCCATTGG-3′.

### Cells

At the Shanghai Institute of Cell Biology, Chinese Academy of Sciences (Shanghai, China), four human GBM cells (U87-MG, A172, U251-MG, and T98G) were purchased, and grown at 37°C with 5% CO_2_ in modified Eagle’s medium (catalog number SH30022.02, HyClone, Logan, UT, USA) supplemented with fetal bovine serum (10%) (catalog number 10100147, Gibco, Carlsbad, CA, USA), penicillin (100 units/mL), and streptomycin (100 g/mL).

### siRNA transfection

GRIK1 siRNA was produced by Ruibo Biotech (Guangzhou, China). The GRIK1 siRNA was transfected into U87-MG and T98G cells by Lipidnano^TM^ Super RNAi Transfection Reagent (cat TL-1001, Biotechnology, Beijing, China). We designed and purchased three siRNAs types to knock down the expression of GRIK1, such as GRIK1 siRNA1: GCUGUGCAGUCUAUUUGCAAU; GRIK1 siRNA2: CCUCACGUGUCAUCCAUCAUU; GRIK1 siRNA3: UCCACUUCUGGAACUACUUUA.

### Cell proliferation assay

In a 96-well plate (cat 713011, NEST, Shanghai, China), cells were plated at a density of 2500 cells per well. 10 μL of Cell counting kit-8 (CCK8) reagent (cat CK04, Dojindo, Kumamoto, Japan) was applied. Absorbance at OD450 was collected at 24, 48, 72, 96, and 120 h. At least three times were given to each test. Using a microplate reader, the absorbance was calculated at a wavelength of 450 nm. Using a microplate reader, the absorbance was calculated at a wavelength of 450 nm.

### Colony formation experiments

In 6-well plate (cat 703001, NEST, Shanghai, China), a hundred cells per well were implanted. After nine days, colonies that had developed were stabilized and tagged with 0.1% crystal violet. Representative colonies (>200 cells/colony) were calculated, and each experiment was carried out three times.

### Wound healing assays

Cells (90% confluence) were seeded in 6-well plates (cat 703001, NEST, Shanghai, China) and rubbed with a 200 µL pipette tip. Healing ability was determined as the percentage change of the initial induced injury width. Each experiment was done in triplicate.

### Transwell and Matrigel-transwell experiments

The upper chambers of 24-well Transwell plates (8-µm pores; BD Biosciences) were seeded with a total of 2 × 10^5^ U87 or T98G cells for invasion or migration, either with or without a thin covering of Matrigel (37°C for 1 h; BD Biosciences). The bottom chamber was added with a complete medium. After overnight incubation, to get rid of any remaining cells, the top layer was cleansed using a sterilized cotton swab. The migrated cells were stained with 0.1% crystal violet (cat C0121-100ml Beyotime Biotechnology, Shanghai, China) and counted. In five randomly chosen fields per well, the number of cells was computed while taking the average into account. The assays were carried out three times.

### Statistical analysis

The statistical analysis was conducted in SPSS (version 17.0, Chicago, USA). When the *p* value (two-sided) was less than 0.05, the results were deemed significant. The Kaplan–Meier and log-rank tests were used to examine the GRIK1 survival curve.

## Results

### GRIK1 is upregulated in recurrent GBM

**Figure 1:**
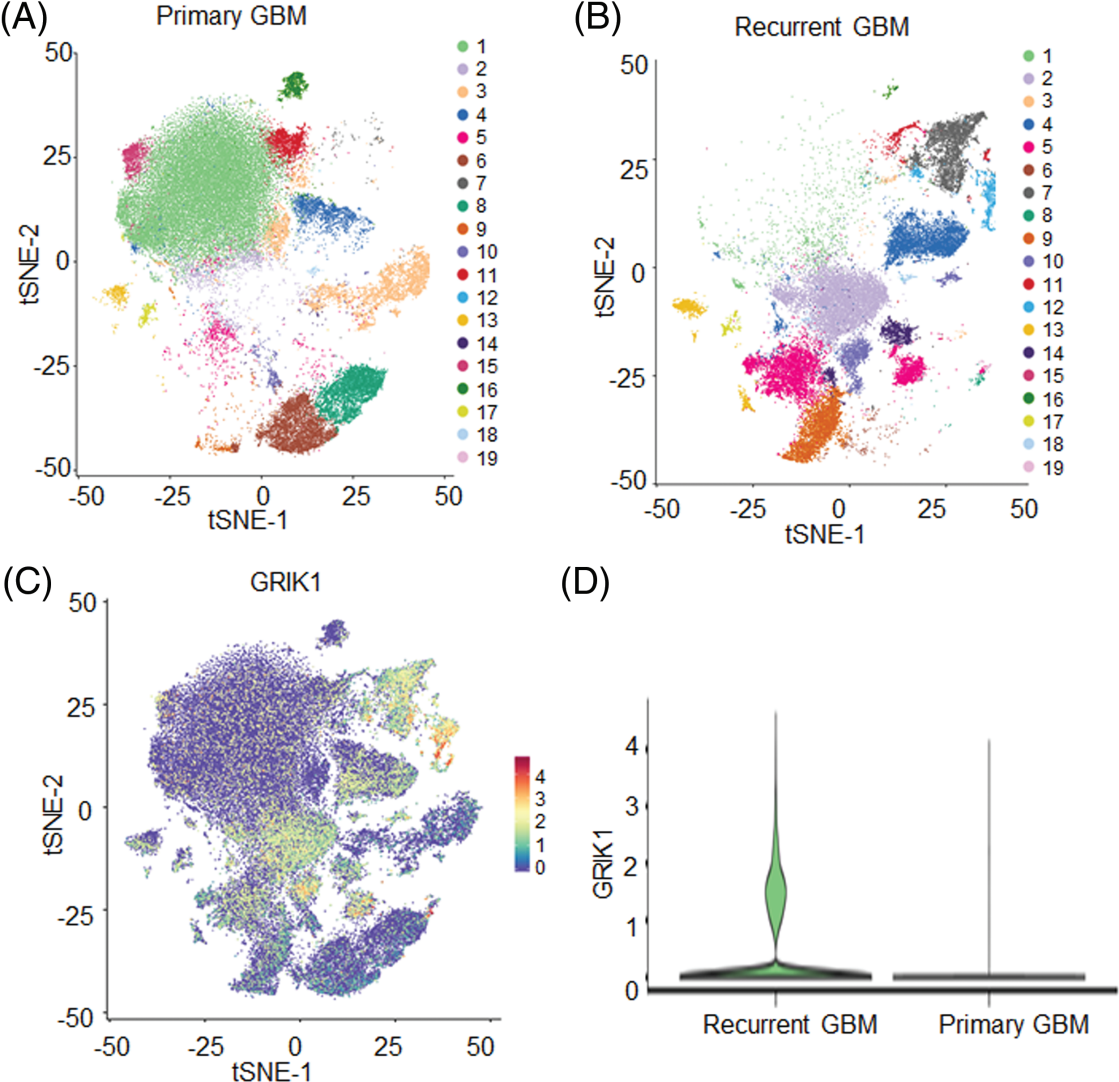
GRIK1 is highly expressed in recurrent GBM. (A, B), tSEN dimensional reduction of GBM cells from primary (n = 3) or recurrent (n = 3) GBM patients. (C), Pattern representing single-cell gene expression of GRIK1 in GBM. Differential colors indicate the expression levels of individual cells. (D), Violin plot shows the expression levels of GRIK1 in recurrent and primary GBM.

The *t*-distributed stochastic neighbor embedding technique split cells into 19 clusters after single-nuclear RNA sequencing (Figs. 1A–1C). GRIK1 was significantly more prevalent in the recurrent group and was found in the majority of clusters (Figs. 1C and 1D**).**

### Compared with normal tissues, GBM has a higher expression of GRIK1

**Figure 2:**
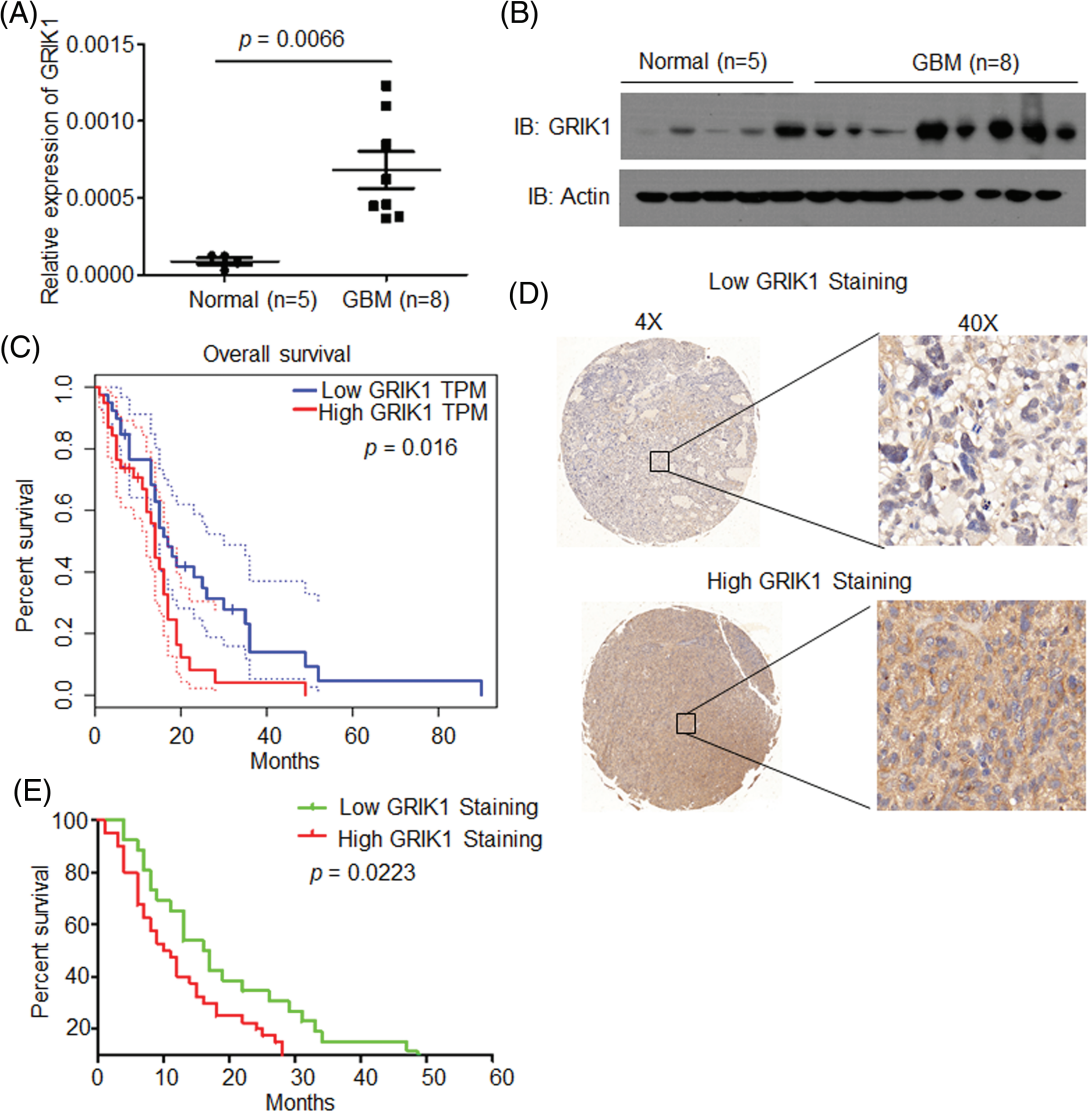
GRIK1 is highly expressed in GBM and is associated with poor prognosis of GBM. (A, B) mRNA and protein level of GRIK1 in GBM tissues (n = 8) or normal brain tissues (n = 5), *p* = 0.0066. (C) Survival curves of patients with GBM displaying low or high expression of GRIK1 in TCGA database, *p* = 0.0016. (D) Representative images showing low or high levels of CHRM3 in GBM samples using immunostaining analysis. (E) Survival curves of GBM patients with high or low levels of GRIK1, *p* = 0.0223.

The levels of GRIK1 in newly collected normal brain tissue (n = 5) and GBM tissue (n = 8) were investigated by RT-qPCR and WB to further support the significance of GRIK1 for GBM prognosis. WB results showed that GRIK1 was significantly more expressed in GBM tissues than in normal tissues (Figs. 2A and 2B).

### GRIK1 is associated with poor prognosis

To investigate the prognostic significance of GRIK1 in GBM, survival analysis was performed in the TCGA database. The results revealed that higher levels of GRIK1 were connected to poor survival in GBM patients significantly (Fig. 2C).

Next, we detected GRIK1 levels in the GBM tissue microarray. Representative staining patterns of GRIK1 in GBM are shown in Fig. 2D. After that, the GBM samples were divided into groups with high and low GRIK1 expression. Patients with high GRIK1 levels had a significantly worse prognosis, according to log-rank tests and Kaplan-Meier analyses (n = 65, *p* < 0.05, Fig. 2E).

Taken together, these findings revealed that GRIK1 was considerably upregulated in GBM and was associated with poor prognosis in both our GBM samples and the TCGA database.

### Knockdown of GRIK1 inhibits GBM cell growth ability

**Figure 3:**
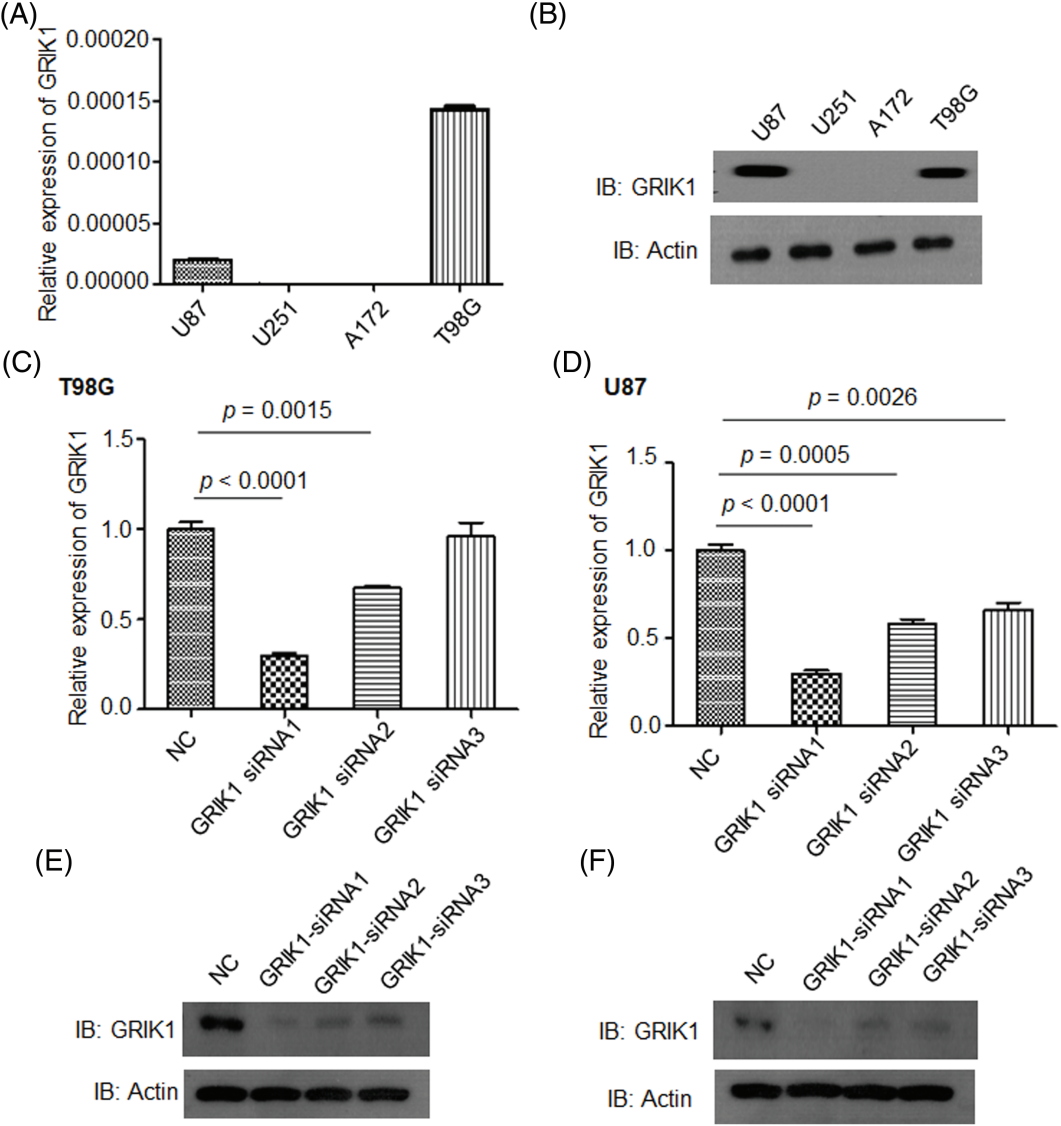
Construction of GRIK1 knockdown in GBM cells. (A, B) Relative levels of GRIK1 mRNA and protein in U87-MG, A172, T98G, and U251-MG cells. (C–F) Relative levels of GRIK1 in T98G (C, E) and U87-MG cells (D, F) with or without GRIK1 knockdown.

To clarify the pathological roles of GRIK1 in GBM development, we first studied the levels of GRIK1 in an array of cell lines. U87-MG and T98G cells had relatively greater levels of GRIK1 than A172 and U251-MG cells, according to RT-qPCR and WB data (Figs. 3A and 3B). We then efficiently knocked down the expression of GRIK1 in U87-MG and T98G cells, and found that GRIK1 siRNA1 was the most efficient sequence, so it was used for the following experiments (Figs. 3C–3F).

**Figure 4:**
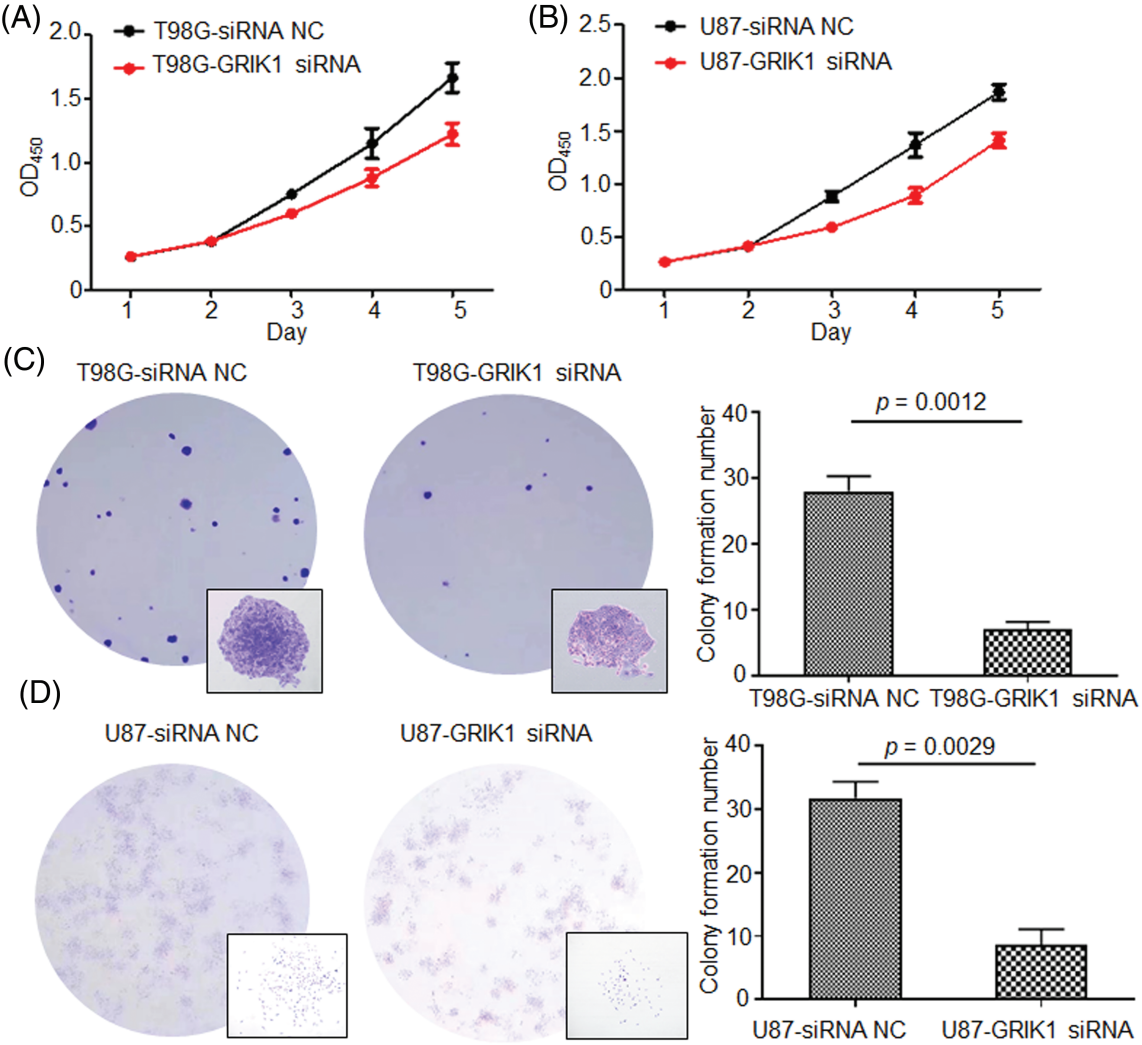
Knockdown of GRIK1 inhibits GBM cell growth ability. (A, B), Proliferation abilities of T98G (A) and U87-MG cells (B) with or without GRIK1 knockdown. (C, D), Colony formation assay of T98G (C) and U87-MG cells (D) with or without GRIK1 knockdown.

The pathological roles of GRIK1 in the proliferation and invasion abilities of GBM were assessed with or without GRIK1 knockdown. CCK8 showed that inhibition of GRIK1 significantly reduced cell proliferation abilities (Figs. 4A and 4B). Moreover, colony formation assay further indicated that GRIK1 knockdown significantly inhibited colony numbers (Figs. 4C and 4D).

### Knockdown of GRIK1 inhibits GBM cell invasion ability

**Figure 5:**
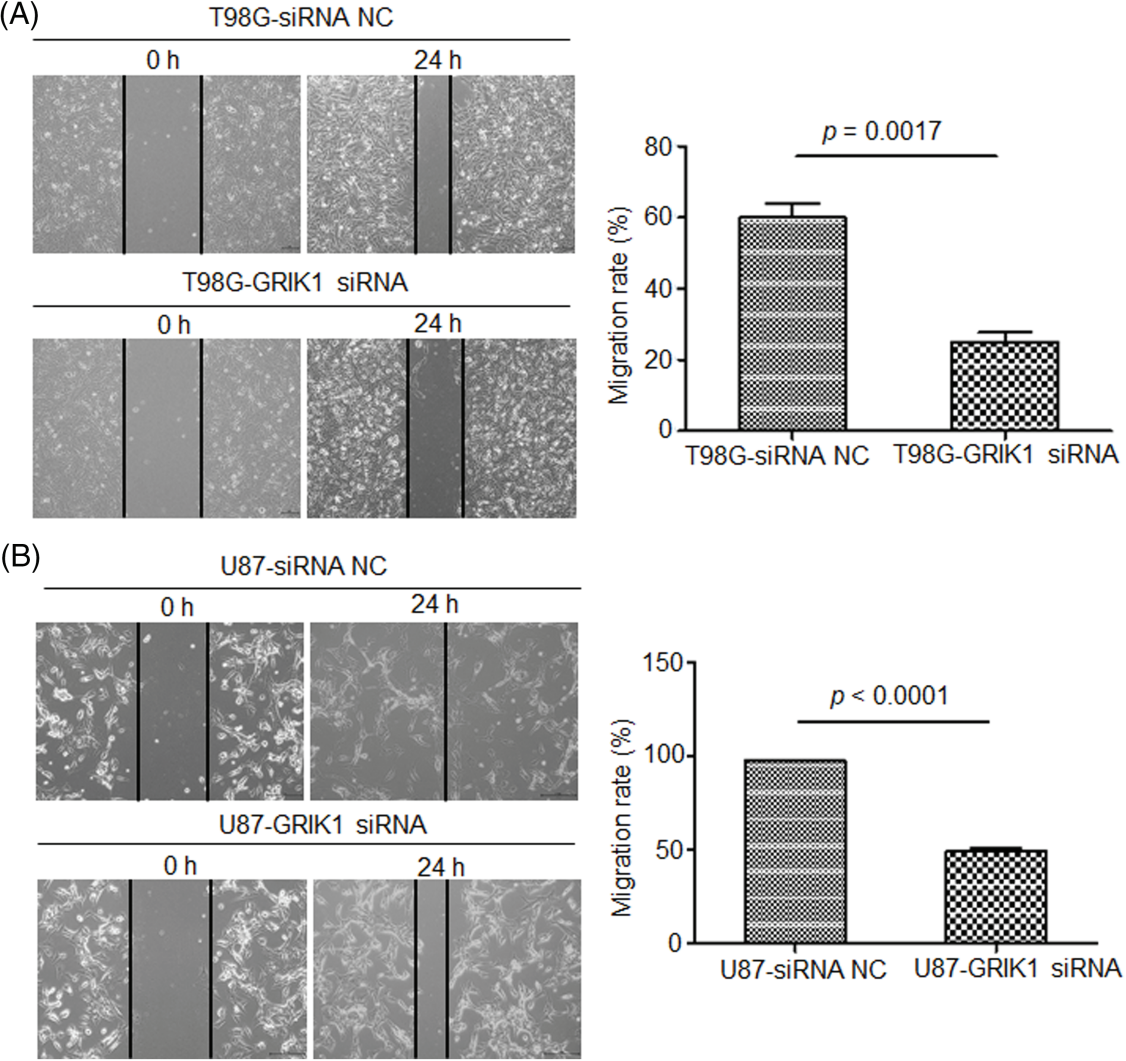
Knockdown of GRIK1 inhibits GBM cell migration ability. (A, B) Relative migration rate of T98G (A) and U87-MG cells (B) after scratching, with GRIK1 inhibition or negative control.

**Figure 6:**
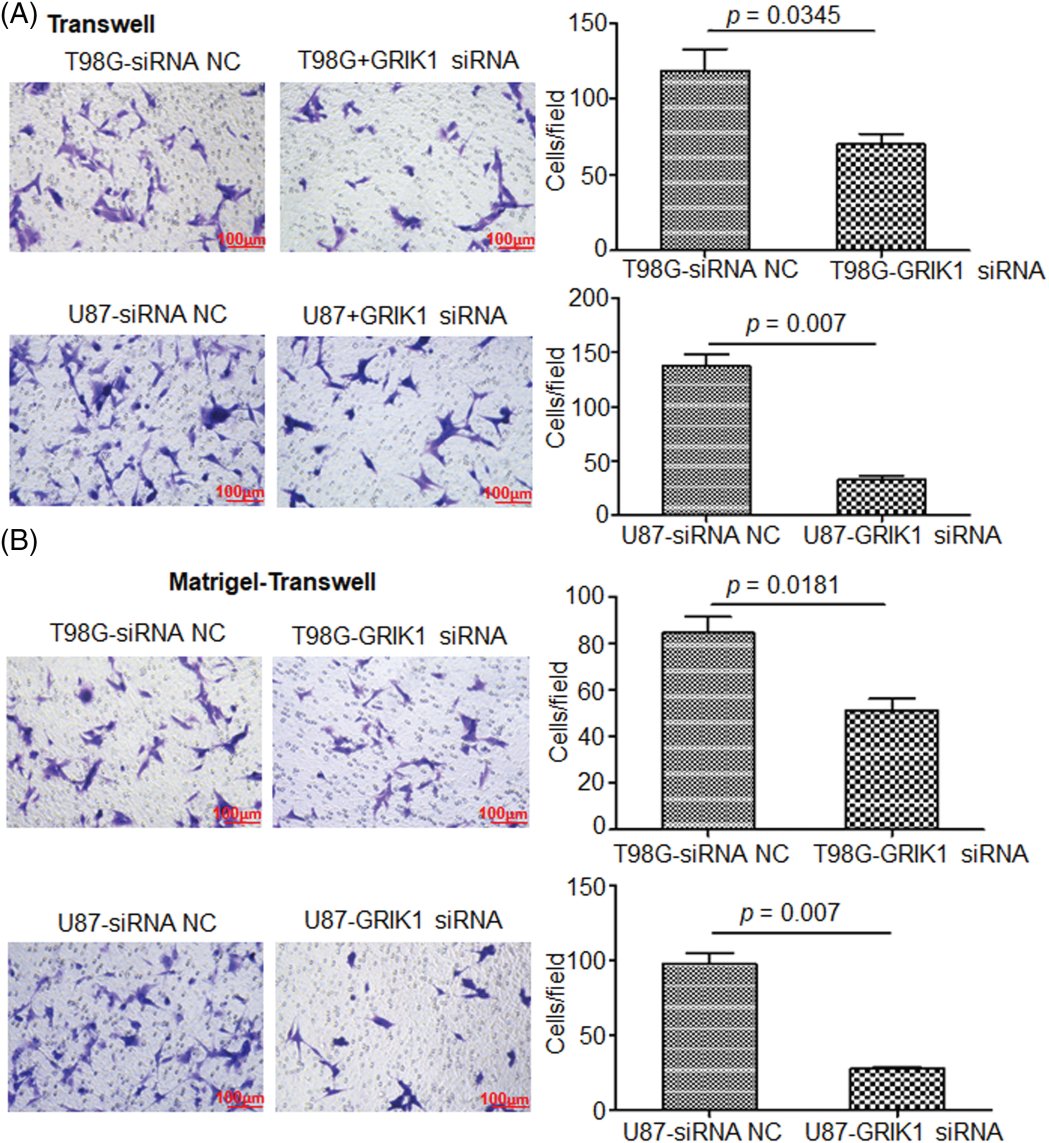
Knockdown of GRIK1 inhibits GBM cell invasion ability. (A, B), The numbers of migrated (A) or invading cells (B) in T98G and U87-MG, with GRIK1 inhibition or negative control.

The scratch assay revealed that GRIK1 knockdown dramatically reduced an ability of GBM cells to migrate (Fig. 5). Further, both the Transwell and Matrigel-Transwell invasion assays revealed that GRIK1 knockdown significantly reduced GBM proliferation and migratory capabilities (Fig. 6).

**Figure 7:**
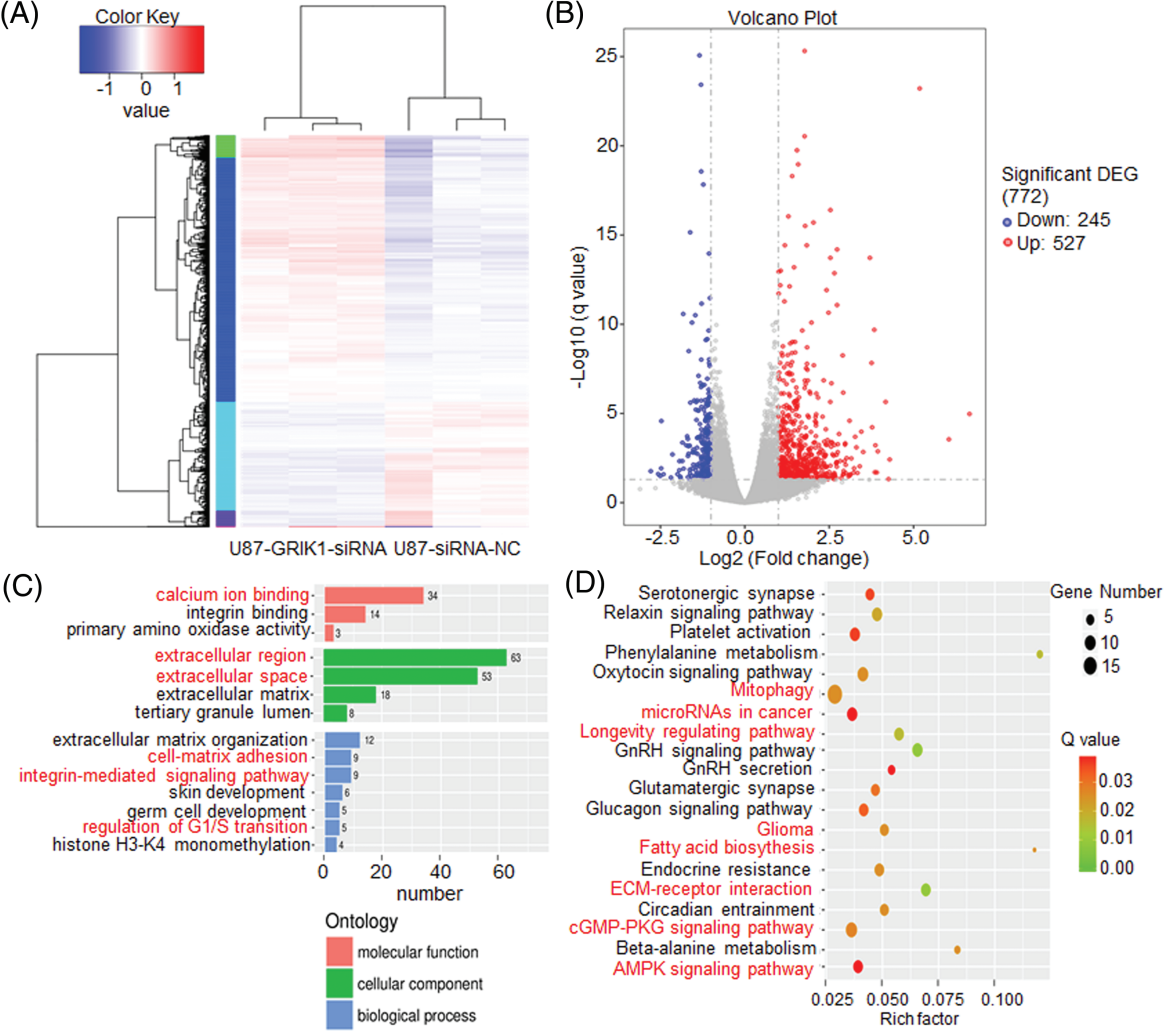
GRIK1 knockdown drastically lowers a variety of traditional disease-related factors. (A, B) Transcriptome sequencing results of U87-MG cells, with or without GRIK1 knockdown. (C, D) GO and KEGG analysis of differentially expressed genes in U87-MG cells, with or without GRIK1 knockdown.

To further investigate the molecular mechanisms of GRIK1 in GBM, transcriptome sequencing was carried out in U87-MG cells with or without GRIK1 knockdown. We identified over 700 genes that were differentially expressed, including 245 downregulated and 527 upregulated genes (Figs. 7A and 7B). Kyoto Encyclopedia of Genes and Genomes (KEGG) and Gene Ontology (GO) analyses showed that most of the differentially expressed genes were associated with cancer-associated pathways, such as the regulation of the G1/S transition, the Adenosine 5′-monophosphate (AMP)-activated protein kinase (AMPK) signaling pathway, the longevity regulation pathway, and microRNAs in cancer (Figs. 7C and 7D).

Together, the current work investigated GRIK1’s function in GBM growth and invasion potential. The levels of GRIK1 were significantly elevated in the GBM and recurrent GBM samples. Additionally, short survival times and poor prognosis were linked to high levels of GRIK1. The ability of GBM cells to develop and invade was decreased after GRIK1 inhibition. Our research indicates that GRIK1 is a new and important element in the development of GBM and may be used as a new biomarker for the detection and treatment of GBM patients.

## Discussion

GBM is the most aggressive and lethal kind of glioma, with the worst prognosis. There have been numerous attempts to create new medications and increase patient survival, but the results have not been sufficiently good [32]. New prognostic markers and targets are essential to improve GBM treatment. Yet, the molecular mechanisms of GBM invasive growth remain largely unknown. In brain regions responsible for learning and memory, GRIK1 is encoded by the GRIK1 gene [17], which is located on chromosome 21q21.1–22.1 [16]. In previous studies, the relationship between GRIK1 and disease has been studied in multiple fields [22,23,27–29,31]. However, GRIK1’s pathogenic roles and mechanisms in GBM are still unknown.

Considering that cancer cells proliferate more quickly and have higher metabolic efficiency than normal cells, glutamate’s participation in the energy cycle is essential in tumor cells and rapidly proliferating cells. Glutamate is an external ligand that can activate various glutamate receptors in the autocrine/paracrine circuit and the signaling pathways involved in the proliferation of cells. The presence of intracellular glutamate also aids in the activation of glutamate receptors, which in turn causes various signals and controls cell growth [20]. Ionic glutamate receptors are involved in the most rapid excitatory synaptic transmission in the central nervous system. These receptors are crucial for the formation of learning and memory, but excitotoxicity caused by excessive activation of glutamate receptors leads to neuronal dysfunction and death. Indeed, excitotoxicity is involved in the pathogenesis of many neurodegenerative diseases with different etiologies, such as Alzheimer’s disease and Parkinson’s disease [33,34]. Single-nuclear RNA sequencing revealed that GRIK1 levels were considerably greater in the recurrent GBM samples than in the primary GBM samples. This suggests that GRIK1 may be strongly related to the prognosis of GBM. To investigate the relevance of GRIK1 in GBM prognosis, the levels of GRIK1 in freshly obtained normal brains (n = 5) and GBM tissues (n = 8) were examined by RT-qPCR and WB. GBM tissues compared with the normal tissues. Furthermore, survival analysis was performed in the TCGA database, and the expression levels of GRIK1 were detected by tissue microarray. We found that lower survival rates in GBM patients were significantly associated with greater levels of GRIK1. There are many molecules associated with GBM prognosis, and we confirmed that GRIK1 is one of them. The latest research has shown that Sprouty4 (SPRY4) is an independent favorable prognostic factor of GBM, and it could suppress GBM invasion by the ERK–ETS–MMP9 axis [32]. In addition, the expression levels of miR-373 and miR-520s, along with CD44, may be useful biological markers of the prognosis of GBM since they work by inhibiting CD44 expression in GBM cells [35].

We conducted several cell function tests to clarify the pathogenic functions of GRIK1 in the development of GBM. First, we selected U87-MG and T98G cells with higher levels of GRIK1 in an array of cell lines by RT-qPCR and WB and then knocked down their expression of GRIK1 efficiently. Meanwhile, we assessed the pathological roles of GRIK1 in the proliferation and invasion abilities of GBM, with or without CHRM3 knockdown. CCK8 showed that inhibition of GRIK1 significantly reduced cell reproduction abilities. The Matrigel-Transwell invasion assay, colony formation investigations, and wound healing assays all demonstrated that the capacity of GBM cells to invade was greatly reduced when GRIK1 was inhibited. The molecular mechanisms of GBM growth and invasion are extremely complex and include many factors, such as lactate dehydrogenases [36], mitogen-activated protein kinase kinase kinase 1(MAP3K1)/c-JUN signaling-axis [37], Hsa_circ_0072309 [38], RNA-binding motif protein 8A (RBM8A) [9], LINC01711 [10],COL1A2 [11], adipocyte enhancer-binding protein 1 (AEBP1) [39], Nucleobindin-2 [40], the cross-talk between Notch1 signaling and CXCL12/CXCR4 system [41], PRL1/USP36/Snail2 axis [42], Zinc finger CCCH-type containing 15 (ZC3H15) [43], circ_0060055 [44], and Rhoj/Rac1/PAK signaling [45], etc.

GRIK1 is associated with many diseases, and it has many modification sites, such as common phosphorylation [46], glycosylation [47], and SUMOylation [48], etc. Therefore, GRIK1 may have great potential for drug research, especially for its relationship with tumors.

Regrettably, specific regulation mechanisms between GRIK1 involved in cancer invasive growth in GBM cells remain unclear, and transcriptional factors regulations after GRIK1 need further studies.

## Supplementary Materials



## Data Availability

All data are available from the corresponding author upon reasonable request. The single nuclear-sequences data has been deposited in the Genome Sequence Archive in National Genomics Data Center, China National Center for Bioinformation/Beijing Institute of Genomics, Chinese Academy of Sciences (GSA-Human: HRA003631) that are publicly accessible at https://ngdc.cncb.ac.cn/gsa-human. The data of transcriptome analysis has been deposited in GEO (accession numbers: GSE242008) that are publicly accessible at https://www.ncbi.nlm.nih.gov/geo/query/acc.cgi?acc=GSE220083.

## Supplementary Materials

The supplementary material is available online at https://doi.org/10.32604/or.2023.043391.
